# Enhancing soil fertility in urban green spaces via cellulolytic microbial-organic synergies

**DOI:** 10.3389/fmicb.2026.1711396

**Published:** 2026-02-05

**Authors:** Zhaofeng Xu, Jiawei Dai, Ning Yang, Yongjie Fan, Xin Shan, Yuting Diao, Xiaocui Pan, Lei Zhao, Jiahui Zhao, Meiqi Ma, Xiang Li, Ming Xiao, Junmin Pei

**Affiliations:** 1 College of Life Sciences, Shanghai Normal University, Shanghai, China; 2 School of Life Sciences, Taizhou University, Taizhou, Zhejiang, China; 3 Shanghai Institute of Quality Inspection and Technical Research, Shanghai, China

**Keywords:** *Bacillus cereus* B9, cellulose degradation, microbial community, nutrient cycling, urban green spaces, vermicompost

## Abstract

Urban green spaces (UGSs) are essential for ecological functioning, yet their soils often suffer from limited nutrient cycling due to the slow decomposition of plant litter. While cellulolytic bacteria can promote litter breakdown by enhancing cellulose degradation, their effectiveness in urban soils remains limited. In this study, we examined whether combining *Bacillus cereus* B9, a cellulolytic strain, with vermicompost could improve litter decomposition and soil quality in UGS soils. A pot experiment was conducted with four treatments: control (CK), B9 alone (B9), vermicompost alone (V), and their combination (VB9). Results showed that the VB9 treatment significantly enhanced litter decomposition, cellulase activity, and nutrient availability compared to either treatment alone. Genome sequencing revealed that B9 carries key cellulase genes, including those encoding endoglucanase and *β*-glucosidase. Enzyme assays confirmed its cellulolytic activity. Co-application also enriched bacterial taxa associated with cellulose degradation, whose abundance was positively correlated with increased soil ammonium and alkali-hydrolyzable nitrogen. B9 likely contributed to ammonium accumulation via the dissimilatory nitrate reduction to ammonium (DNRA) pathway. Non-targeted metabolomics further indicated enhanced nitrogen and carbon metabolic activity in VB9 soils. These findings support the synergistic effect of microbial inoculants and organic amendments in improving organic matter turnover and nutrient cycling in urban soils. Further research is needed to assess this strategy’s long-term efficacy and ecological impact under field conditions.

## 1 Introduction

Urban green spaces (UGSs) are crucial in enhancing biodiversity, regulating microclimates, and improving air quality (Salmond et al., 2016; Paudel and States, 2023). However, UGS soils often suffer from slow organic matter turnover, nutrient deficiencies, and low microbial activity, which limit plant growth and ecosystem functions (Lan et al., 2019; Mónok et al., 2021; Sun et al., 2023). A key challenge in UGS soil restoration is the accumulation of plant litter with high cellulose and lignin content, which impedes nutrient cycling and organic matter turnover (Pavao-Zuckerman and Coleman, 2005; Huang et al., 2021; Wu et al., 2022). Therefore, effective soil restoration strategies should improve nutrient availability and accelerate lignocellulose degradation to enhance long-term soil productivity.

Vermicompost is widely recognized as an effective organic amendment for improving soil fertility due to its rich nutrient content and beneficial effects on soil structure (Joshi et al., 2015; Domínguez et al., 2019). It enhances soil porosity, aggregate stability, and aeration while supplying readily available macro- and micronutrients (Aksakal et al., 2016; Zhou et al., 2022). Recent studies further indicate that fresh vermicompost can contribute to the degradation of cellulose and lignin, because it contains diverse cellulolytic and ligninolytic microorganisms and their extracellular enzymes (Karthika et al., 2020; Hřebečková et al., 2024). However, its effectiveness in decomposing highly recalcitrant plant litter remains variable, largely due to the substantial decline in microbial biomass and enzyme activity during vermicompost aging—particularly for cellulase and *β*-glucosidase (Aira et al., 2007). As microbial activity is a major determinant of lignocellulose breakdown (Bai et al., 2024), the capacity of vermicompost alone to process intact litter is often limited. These constraints highlight the need to integrate functional microbial inoculants with vermicompost to enhance decomposition efficiency and improve soil fertility in UGSs.

Microbial inoculants have been widely studied for their potential to enhance soil quality by promoting nutrient solubilization and organic matter turnover (Sammauria et al., 2020; Jeewani et al., 2021). While most research has focused on phosphate-solubilizing and nitrogen-fixing bacteria to improve nutrient supply in agricultural soils, microbial contributions to cellulose degradation in UGS soils remain largely unexplored (Kumar et al., 2016). Additionally, most studies have examined either vermicompost or microbial inoculants independently, whereas their combined effects, particularly in cellulose-rich urban soils, have rarely been investigated. The decomposition of structural organic compounds is often constrained in UGS soils due to limited bioavailable carbon and nutrients (Yang et al., 2014; Gómez-Brandón et al., 2022). Therefore, microbial inoculation alone may not be sufficient to accelerate litter decomposition and improve soil fertility in UGS environments, highlighting the need for strategies that combine microbial and organic amendments.

Among microbial inoculants, cellulolytic bacteria play a crucial role in litter decomposition by producing cellulases that break down complex plant polymers into simpler organic compounds (Wang et al., 2022). However, their activity and efficiency in soil environments often depend on additional nutrient sources that sustain microbial metabolism (Khare and Arora, 2015). By supplying labile carbon and essential nutrients, vermicompost could provide a suitable environment for cellulolytic bacteria to thrive and maximize their enzymatic functions (Song et al., 2015; Tumbure et al., 2023). Thus, we hypothesize that the co-application of *B. cereus* B9-a previously isolated cellulolytic strain-and vermicompost will synergistically enhance cellulose degradation, enrich cellulolytic microbial taxa, and improve soil nutrient availability, ultimately promoting soil fertility in UGSs.

To test this hypothesis, we conducted a pot experiment comparing four treatments: control (CK), *B. cereus* B9 inoculation (B9), vermicompost addition (V), and co-application of B9 and vermicompost (VB9). We examined the effects of these treatments on litter decomposition rates, cellulase activity, microbial community composition, and soil nutrient cycling. This study aims to elucidate how microbial inoculation and organic amendments interact to accelerate organic matter decomposition and enhance UGS soil restoration. Our findings will contribute to developing more effective and sustainable soil management strategies for urban ecosystems.

## 2 Materials and methods

### 2.1 Soil, litter, and vermicompost

Topsoil (0–15 cm) was collected from green spaces at Shanghai Normal University (121°25’29”E, 31°10’8”N), sieved through a 2-mm mesh to remove plant debris, and homogenized before use. The soil was characterized as silty loam, and its physicochemical properties (Supplementary Table 1), including pH, alkali-hydrolyzable nitrogen (AN), available phosphorus (AP), and available potassium (AK), were measured using standard methods (Liu et al., 2024). Fallen leaf litter was collected from the same site in November 2023, air-dried, and analyzed (Sjöberg et al., 2004) for cellulose (24%), hemicellulose (17%), and lignin (14%) content. Before the experiment, the litter was cut into 1 cm fragments to ensure consistency in decomposition assays. Commercial vermicompost (organic matter ≥ 40%, N + P₂O₅ + K₂O ≥ 6%) was purchased from Shanghai Wenxing Biotechnology Co., Ltd., China, and was sieved (2 mm) and homogenized before incorporation into the soil.

### 2.2 Bacterial strain and genome sequencing

We isolated eight candidate bacterial strains from humus-rich forest soil using carboxymethyl cellulose (CMC) plates stained with Congo red to visualize cellulolytic halos (Supplementary Figure 1). To compare their functional potential, all isolates were evaluated for a suite of plant growth–promoting and decomposition-related traits. Specifically, cellulase activity was determined following the method of Luciano Silveira et al. (2012); phosphorus and potassium solubilization abilities were assessed according to Yang and Yang (2020); biofilm formation was measured as described by Hussain and Oh (2017); and production of indole-3-acetic acid (IAA) was quantified using the protocol of Guardado-Fierros et al. (2024). Each isolate was assigned a composite functional score based on its performance across these assays (Supplementary Table 2), with detailed results shown in Supplementary Figure 2. Strain B9 achieved the highest score (16.39) and was therefore selected for subsequent genomic and functional characterization.

Strain B9 was cultured in LB broth at 28 °C, 200 rpm, and harvested during the logarithmic phase for inoculation. Whole-genome sequencing was performed using PacBio Sequel IIe and Illumina platforms. Gene function annotation was conducted using COG, CAZy, Swiss-Prot, and KEGG databases to identify genes involved in cellulose degradation (endoglucanase and *β*-glucosidase) and nitrogen metabolism.

### 2.3 Pot experiment and sampling

A 90-day pot experiment was conducted under controlled conditions (28 °C, 50% water-holding capacity). Each pot (20 cm × 20 cm × 15 cm) contained 2 kg of soil and was assigned to one of four treatments (*n* = 5 per treatment): CK (control, no B9 or vermicompost), B9 (Inoculate with B9 bacterial suspension at OD_600_ = 0.8), V (vermicompost amendment, 5% w/w), and VB9 (co-Inoculation B9 with vermicompost). Each pot received 2 g of pretreated litter enclosed in 60-mesh nylon bags (6 × 7 cm), which were buried at the soil surface to simulate decomposition. After 90 days, soil surrounding the litter was collected for further analyses, including soil cellulase activity (stored at 4 °C), DNA extraction and amplicon sequencing (stored at −80 °C), and chemical analysis (air-dried).

To assess litter decomposition, we quantified the litter decomposition rate constant (*k*) and the degradation rates of cellulose, hemicellulose and lignin. The decomposition rate constant (*k*, d^−1^) was calculated using the single exponential model (Yue et al., 2016):


$$\ln(\frac{M_{t}}{M_{0}})=-\mathrm{kt}$$

Where *M_t_* is the remaining litter dry mass at sampling time *t*, and *M_0_* is the initial litter dry mass.

To further evaluate the loss of major litter chemical components, the remaining mass (*R_t_*) and degradation rate (*D*) of cellulose or hemicellulose or lignin were calculated as follows:


$$R_{t}=M_{t}\times C_{t}$$

$$D(\%)=\frac{R_{0}-R_{t}}{R_{0}}\times 100\%$$

Where *M_t_* is the remaining litter dry mass at sampling time *t*; *C_t_* is the concentration of cellulose or hemicellulose or lignin at sampling time *t*; *R_t_* indicates the remaining mass of cellulose or hemicellulose or lignin at the sampling time *t*, *R_0_* is the initial mass of cellulose or hemicellulose or lignin.

### 2.4 Soil microbial and functional analysis

#### 2.4.1 Microbial community analysis

Total genomic DNA was extracted from soil using the TIANamp Soil DNA Kit (TIANGEN, Beijing, China), followed by amplification and sequencing of the V3–V4 region of the bacterial 16S rRNA gene using Illumina MiSeq (Xu et al., 2016). Raw sequences were processed using DADA2 to generate Amplicon Sequence Variants (ASVs), with taxonomic classification, *α*-diversity indices (Shannon, Ace), and *β*-diversity (Bray-Curtis distance, NMDS) analyzed using QIIME2. Functional predictions were conducted using PICRUSt2 for bacteria.

#### 2.4.2 Soil cellulase activities and nitrogen content

Soil cellulase activity was determined by the 3,5-dinitrosalicylic acid (DNS) reducing sugar assay. Fresh soil (5 g) was treated with toluene for 15 min. The dry weight basis was determined from the moisture content of a parallel sample dried at 105 °C. For the assay, 5 mL of 1% (w/v) carboxymethyl cellulose (CMC-Na) solution and 5 mL of acetate buffer (pH 5.5) were added. A control received 10 mL of buffer only. After incubation at 37 °C for 3 days, reducing sugars in the supernatant were quantified by reaction with DNS reagent and measuring absorbance at 540 nm. The activity, corrected against the control and calculated on a dry soil basis, is expressed as mg glucose equivalent released per gram of dry soil per day (mg glucose g^−1^ soil d^−1^).

Soil nitrate (NO_3_^−^-N) and ammonium (NH_4_^+^-N) concentrations were determined by extraction with 2 M KCl. Fresh soil (5 g) was shaken with 30 mL of KCl solution in a 500 mL polyethylene bottle for 1 h, followed by centrifugation at 3000 r/min for 10 min to obtain a clear supernatant. The concentrations of NH_4_^+^-N and NO_3_^−^-N in the supernatant were measured using commercial assay kits (NH_4_^+^-N: G0425F; NO_3_^−^-N: G0426F; Suzhou Grace Biotechnology Co., Ltd., China) following the manufacturer’s protocols. The dry weight of the soil was determined from the moisture content of a parallel sample dried at 105 °C to constant weight. The concentrations are expressed as milligrams of NH_4_^+^-N or NO_3_^−^-N per kilogram of dry soil (mg kg^−1^).

#### 2.4.3 Metabolomic analysis

The LC–MS/MS analysis of the sample was conducted on a Thermo UHPLC-Q Exactive HF-X system equipped with an ACQUITY HSS T3 column (100 mm × 2.1 mm i.d., 1.8 μm; Waters, United States). The mobile phases consisted of 0.1% formic acid in water: acetonitrile (95:5, v/v) (A) and 0.1% formic acid in acetonitrile: isopropanol: water (47.5:47.5:10, v/v) (B). The flow rate was set at 0.40 mL/min, and the column temperature was maintained at 40 °C.

For MS conditions, an ESI source in both positive and negative modes was used; the source temperature was 425 °C; the sheath gas and auxiliary gas were set at 50 arb and 13 arb, respectively; the IS voltage was -3500 V for negative mode and 3,500 V for positive mode; the normalized collision energy (NCE) was varied at 20–40-60 V; the full MS resolution was 60,000, and the MS/MS resolution was 7,500; data-dependent acquisition (DDA) mode was employed; and the m/z range was set from 70 to 1,050.

### 2.5 Functional characterization of B9

B9 cellulolytic activity was evaluated in Modified Mandels medium supplemented with CMC-Na (1%, w/v). The strain was cultured at 28 °C and 200 rpm for 7 days, and crude enzyme extracts were collected daily by centrifugation. Enzyme activity assays were conducted using different substrates: CMC-Na (2%, w/v) for endoglucanase (CMCase), Avicel (1%, w/v) for exoglucanase (Avicelase), p-nitrophenyl-*β*-D-glucopyranoside (p-NPG, 1%, w/v) for β-glucosidase, and Whatman No. 1 filter paper strips (50 mg; 0.5 × 2.0 cm) for filter paper activity (FPA). Reaction mixtures were incubated at 50 °C, and enzyme activities were determined according to Palit and Das (2024). For CMCase, Avicelase, and FPA, one unit (U) of enzyme activity was defined as the amount of enzyme releasing 1 μmol of reducing sugars (expressed as glucose equivalent) per minute, while β-glucosidase activity was defined as the amount of enzyme producing 1 μmol of p-nitrophenol per minute. All enzyme activities were expressed as units per milliliter of crude enzyme extract (U mL^−1^).

### 2.6 Multivariate statistical analysis

The bar graphs and box plots were generated using GraphPad Prism 9.5.1 (Microsoft Windows, USA). Statistical analyses were conducted using SPSS Statistics 25.0 software (IBM, United States). OmicShare’s online analysis platform was employed to produce heatmaps.

To assess microbial community composition differences among treatments, non-metric multidimensional scaling (NMDS) was performed using Bray-Curtis distance matrices based on genus abundance data. The NMDS analysis was conducted in R (vegan package) with two dimensions (k = 2), and stress values were evaluated to determine the goodness-of-fit of the ordination (stress < 0.2 was considered acceptable). PERMANOVA (permutational multivariate analysis of variance, 999 permutations) was used to test for significant differences in microbial community composition among treatments.

Redundancy Analysis (RDA) was used to examine relationships between soil environmental factors and microbial community composition, where explanatory variables included pH, AN, AP, AK, NO₃^−^-N, and NH₄^+^-N, and response variables consisted of ASV and genus abundance matrices from 16S sequencing. Statistical significance was determined using Monte Carlo permutation tests (999 permutations), and RDA plots were generated using Canoco 5.0.

## 3 Results

### 3.1 Co-application of B9 and vermicompost enhances litter decomposition

**Figure 1:**
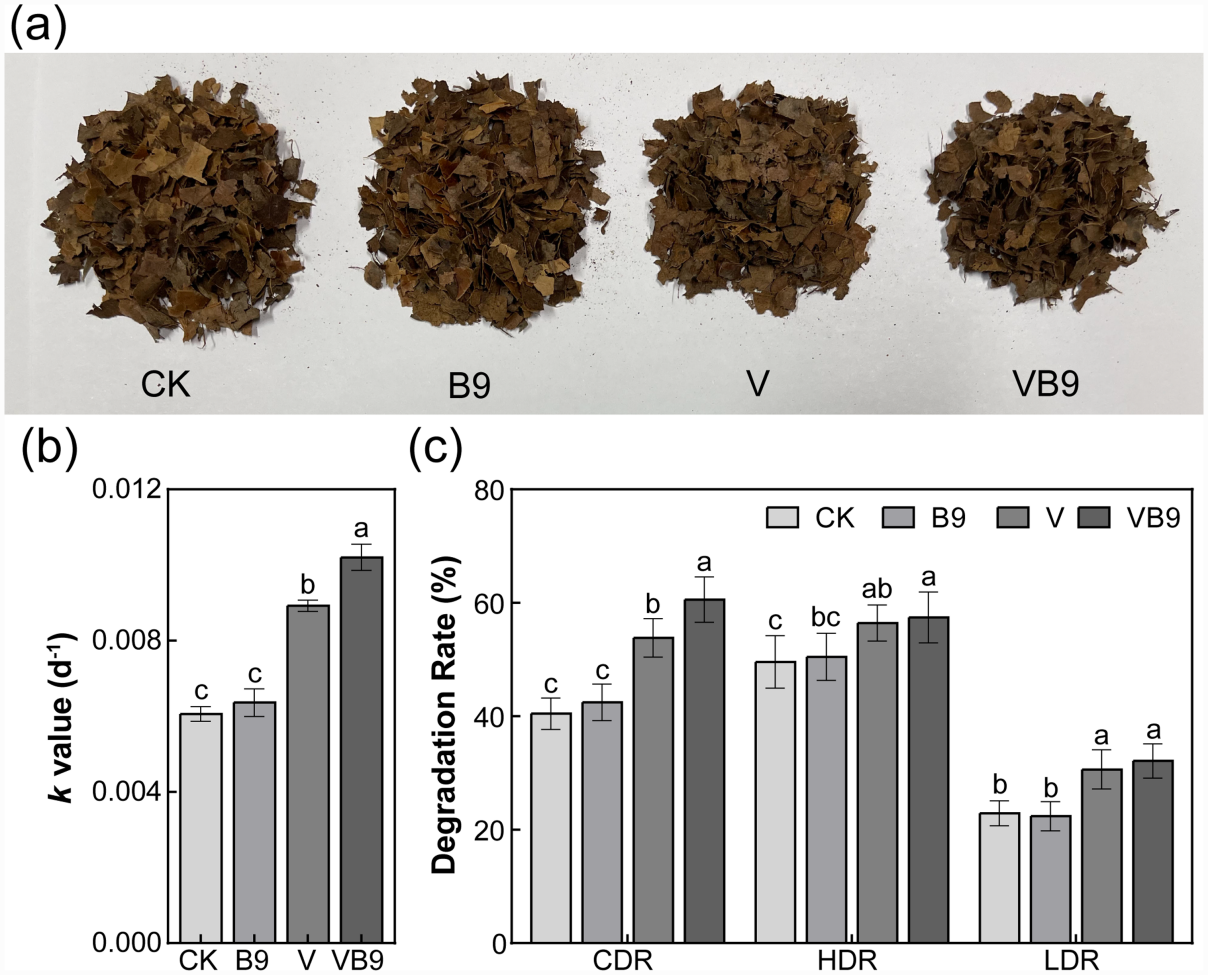
Effects of different treatments on litter decomposition. Representative litter images **(a)**, decomposition rate constant *k* **(b)**, and degradation rates of cellulose, hemicellulose, and lignin **(c)** under four treatments: CK (control), B9 (*B. cereus* B9), V (vermicompost), and VB9 (combined B9 and vermicompost). CDR, cellulose degradation rate; LDR, lignin degradation rate; HDR, hemicellulose degradation rate. Data are shown as means ± SD (*n* = 5). Different letters indicate significant differences (*p* < 0.05, one-way ANOVA).

The decomposition of litter differed significantly among treatments. The VB9 treatment exhibited the highest decomposition rate constant (*k*), followed by V, B9, and CK (Figures 1a,b). Cellulose degradation was notably enhanced in the B9-inoculated treatments, with the VB9 group showing significantly greater cellulose breakdown compared to V (Figure 1c; *p* < 0.05). Linear regression analysis indicated that, among the groups with enhanced decomposition, the cellulose degradation rate was positively correlated with overall litter mass loss (Supplementary Table 3).

### 3.2 Genomic and enzymatic basis for cellulose degradation by B9

**Figure 2:**
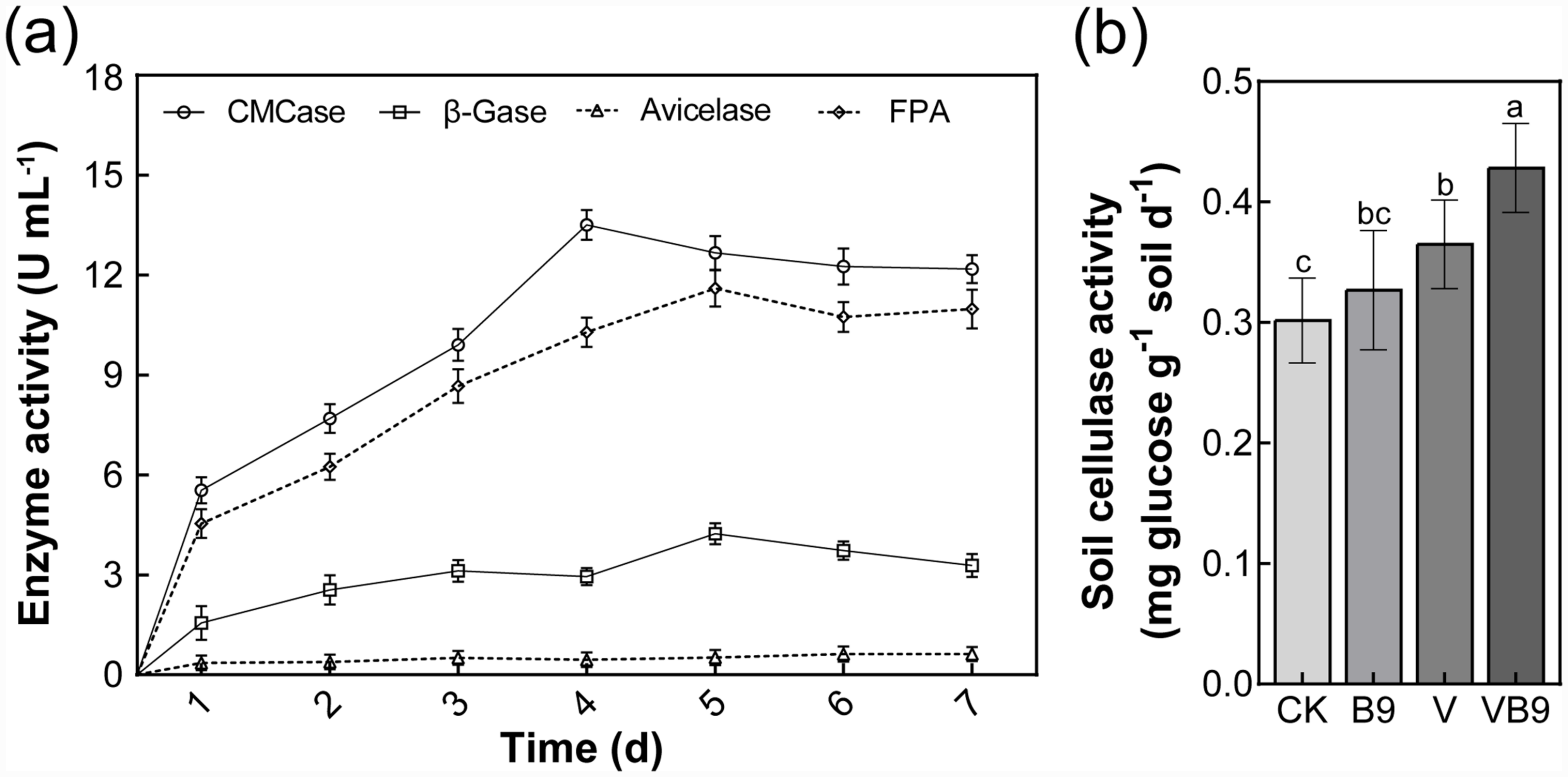
Cellulolytic enzyme activities of *B. cereus* B9 and corresponding soil responses. **(a)** Activities of carboxymethyl cellulase, β-glucosidase, avicelase, and filter paper activity in B9 fermentation broth over 7 days. **(b)** Soil cellulase activity under different treatments. This unit represents the amount of glucose released per gram of dry soil per day, calculated based on a 3-day incubation in the cellulase activity assay. Values represent means ± SD (*n* = 5). Significant differences are indicated by different letters (*p* < 0.05, one-way ANOVA).

Whole-genome sequencing confirmed that strain B9 belongs to *Bacillus cereus* (Supplementary Figure 3). Annotation of carbohydrate-active enzymes revealed the presence of GH1 and GH8 family genes encoding *β*-glucosidase and endoglucanase, respectively (Supplementary Figure 4, Supplementary Table 4). These functional genes were consistent with enzyme assays, where fermentation supernatants showed measurable activity of β-glucosidase, carboxymethyl cellulase (CMCase), and filter paper activity (FPA), reaching maximum values of 4.24, 13.51, and 11.60 U mL^−1^, respectively (Figure 2a). In soil, cellulase activity increased under B9 inoculation, particularly in the VB9 treatment, indicating the expression of these enzymes *in situ* (Figure 2b).

### 3.3 Effects on soil nutrient availability and nitrogen transformation

**Figure 3:**
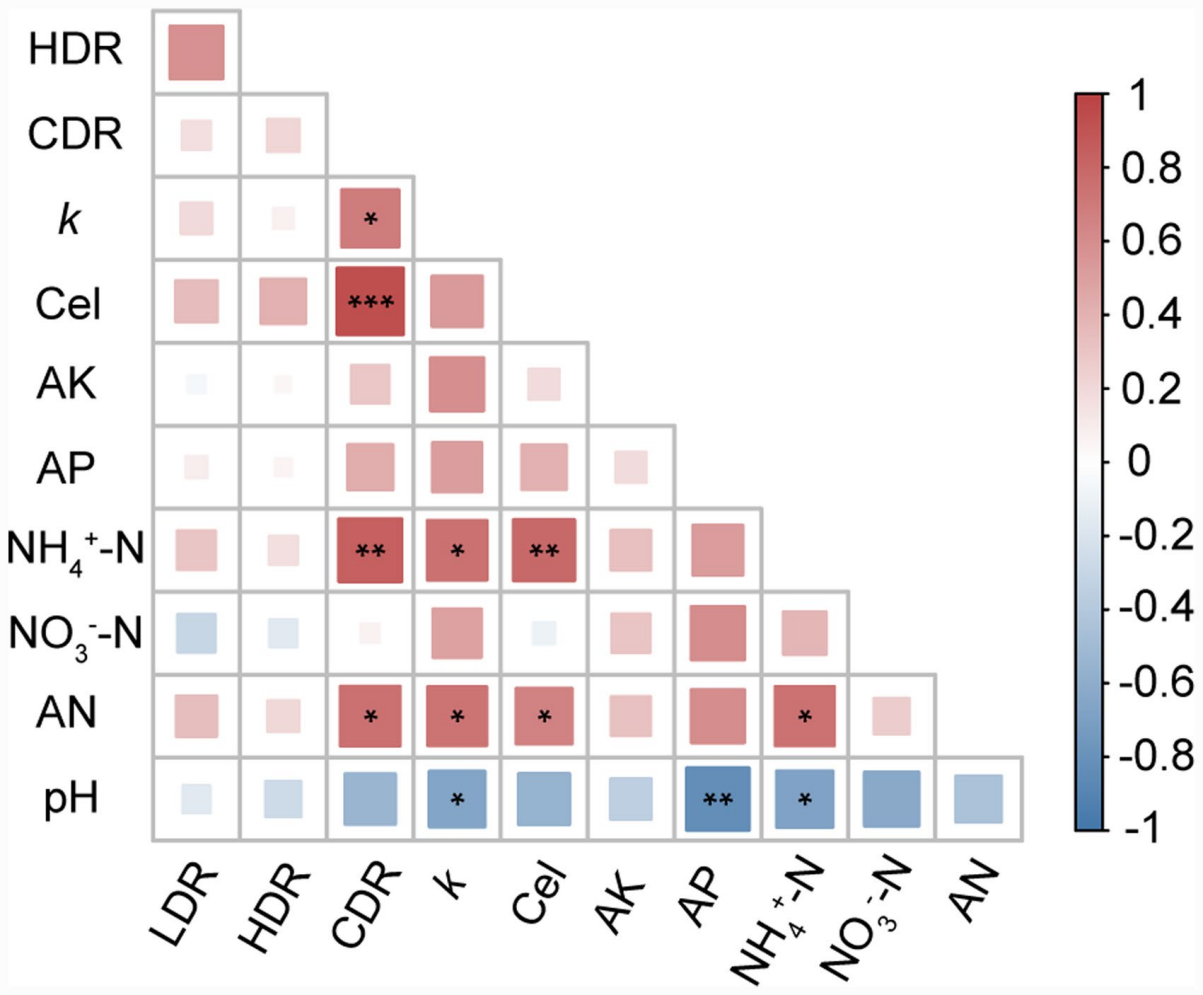
Spearman correlations between litter decomposition parameters and soil properties. Correlation heatmaps between soil physicochemical parameters and litter decomposition metrics in vermicompost-amended soils with or without B9 inoculation. Color intensity reflects the strength and direction of correlations; symbol size indicates |r| values. CDR, Cellulose degradation rate; LDR, lignin degradation rate; HDR, hemicellulose degradation rate; Cel, soil cellulase activity. *p < 0.05, ** < *p* < 0.01, *** *p* < 0.001.

Compared to the V treatment, VB9 significantly improved soil nutrient parameters, including increases of 21.4% in alkali-hydrolyzable nitrogen (AN) and 59.9% in ammonium nitrogen (NH₄^+^-N), along with elevated available phosphorus (AP) and a reduction in soil pH (Table 1). Genome annotation identified multiple genes involved in the dissimilatory nitrate reduction to ammonium (DNRA) pathway (Supplementary Table 5), including *nar*G, *nar*H, *nar*J, *nar*I, *nir*B, and *nir*D (Cai et al., 2018; Huang et al., 2020; Pandey et al., 2020). Cultivation in a denitrification medium verified this potential, with NH₄^+^-N accumulating as nitrate nitrogen (NO₃^−^-N) decreased (Supplementary Figure 5). Spearman correlation analysis indicated positive relationships between AN, NH₄^+^-N, and the litter decomposition and cellulose degradation rates (Figure 3).

**Table 1:** The chemical properties of soil under different treatments.

Treatments	pH	AN (mg kg^−1^)	NO_3_^—^N (mg kg^−1^)	NH_4_^+^-N (mg kg^−1^)	AP (mg kg^−1^)	AK (mg kg^−1^)
CK	8.18 ± 0.02^a^	43.54 ± 1.55^c^	20.74 ± 0.53^b^	0.96 ± 0.07^c^	14.96 ± 0.56^d^	165.44 ± 5.39^b^
B9	8.16 ± 0.02^a^	42.96 ± 1.91^c^	20.60 ± 0.41^b^	1.04 ± 0.07^c^	17.14 ± 0.59^c^	153.24 ± 2.74^c^
V	7.94 ± 0.05^b^	76.06 ± 2.54^b^	37.88 ± 1.19^a^	1.87 ± 0.11^b^	23.96 ± 0.53^b^	216.74 ± 5.11^a^
VB9	7.87 ± 0.04^c^	92.34 ± 3.18^a^	39.67 ± 1.80^a^	2.99 ± 0.20^a^	25.94 ± 1.23^a^	222.48 ± 6.68^a^

pH, potential of hydrogen; AN, alkali-hydrolysable nitrogen; NO_3_^−^-N, nitrate nitrogen; NH_4_^+^-N, ammonium nitrogen; AP, available phosphorus; AK, available potassium. Treatments: CK (control), B9 (*B. cereus* B9), V (vermicompost), VB9 (combined B9 and vermicompost). Data are represented as means ± SD (*n* = 5). Different letters within the same column denote significant differences at *p* < 0.05 (one-way ANOVA).

### 3.4 Microbial community shifts under b9 and vermicompost treatments

**Figure 4:**
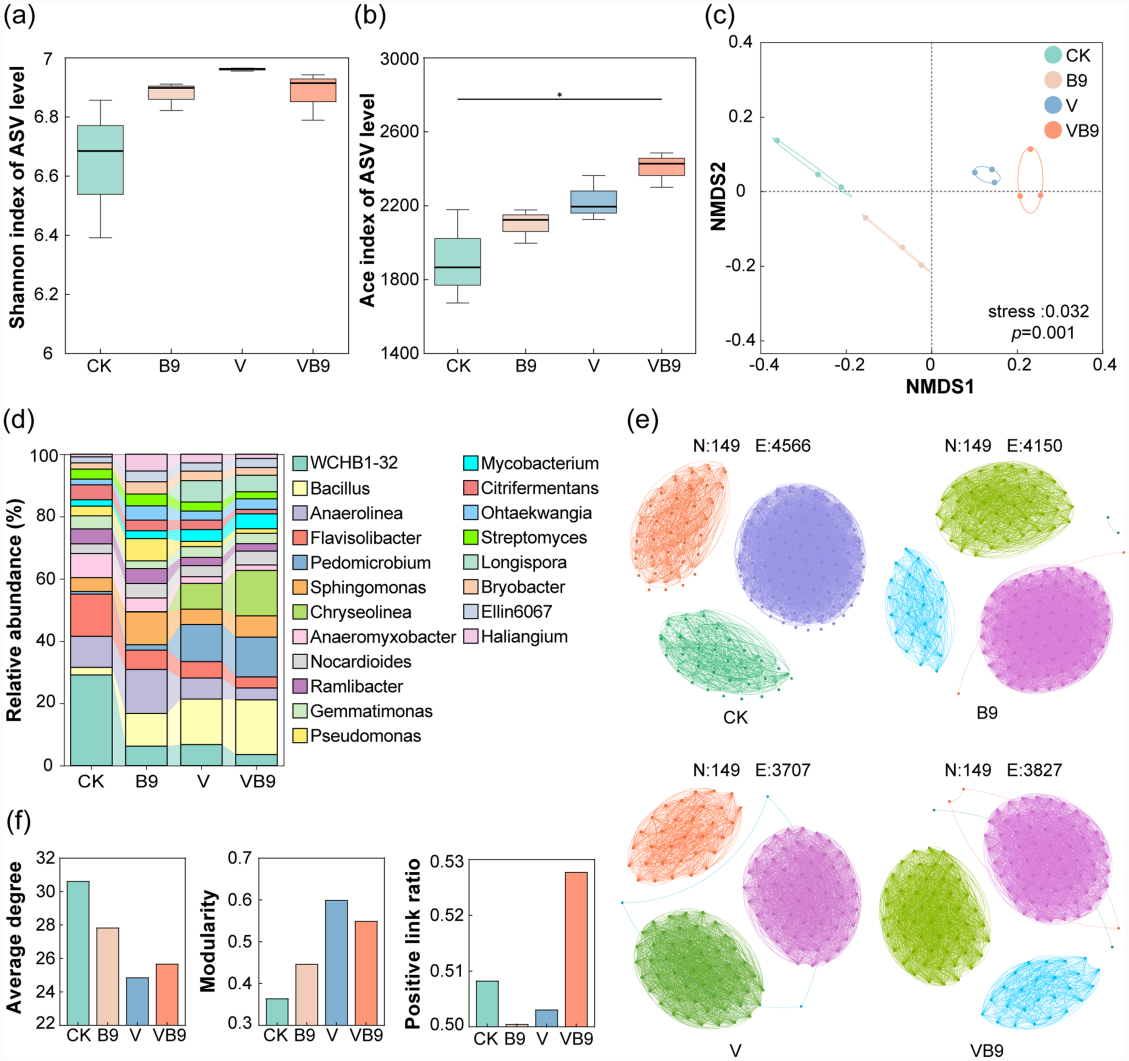
Impacts of treatments on soil bacterial community structure. **(a,b)** *α*-Diversity indices (Shannon and ACE); **(c)** NMDS ordination based on Bray-Curtis distances (stress < 0.2); **(d)** genus-level composition profiles; **(e)** co-occurrence networks of bacterial taxa; **(f)** network characteristics including average degree, modularity, and positive edge ratio. Differences tested via Kruskal-Wallis test (* *p* < 0.05).

The ACE index showed increased bacterial richness in VB9 compared to CK, although Shannon index differences were insignificant (Figures 4a,b). NMDS ordination revealed distinct microbial communities across treatments, with VB9 most divergent from CK (Figure 4c). Taxonomic profiling highlighted greater relative abundances of *Bacillus* and *Sphingomonas* in the B9 and VB9 groups (Figure 4d). These genera were positively correlated with AN, NH₄^+^-N, AP, and AK (Supplementary Figure 6), and *Sphingomonas* abundance showed strong correlation with decomposition metrics (Supplementary Figure 7). Network analysis suggested that VB9 simplified the co-occurrence network while increasing node connectivity (Figures 4e,f), and FAPROTAX predictions indicated enhanced functions related to nitrate reduction and cellulolysis (Supplementary Figure 8).

### 3.5 Alterations in soil metabolite profiles

**Figure 5:**
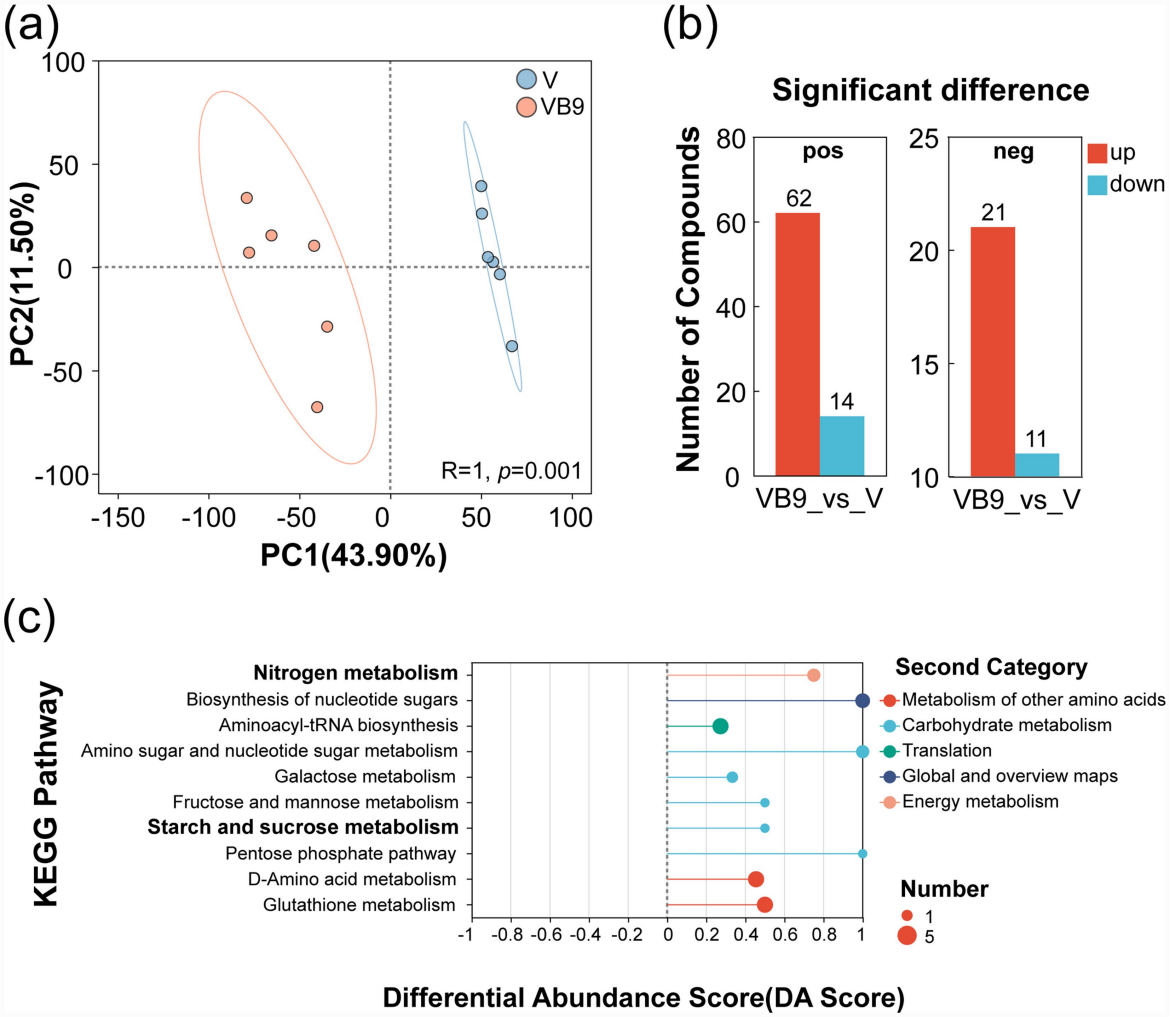
Soil metabolome changes induced by B9 under vermicompost amendment. **(a)** Principal component analysis (PCA) showing metabolomic differences between VB9 and V groups; **(b)** Count of significantly altered metabolites; **(c)** Pathway enrichment (DA score) highlighting shifts in nitrogen, starch, and sucrose metabolism. Dot size reflects the number of differential metabolites; line length represents DA score magnitude.

Non-targeted metabolomic profiling revealed that VB9 treatment shifted overall soil metabolic activity relative to V (Figure 5a). Among the detected metabolites, 62 cationic and 21 anionic compounds were upregulated in VB9, while 14 cationic and 11 anionic compounds were downregulated (Figure 5b). DA-score analysis showed enrichment in pathways associated with starch and sucrose metabolism and nitrogen metabolism (Figure 5c). Notably, trehalose, L-glutamic acid, L-glutamate, and glutamate levels were elevated in VB9 (Supplementary Figure 9), supporting metabolic activity aligned with cellulose degradation and nitrogen turnover.

## 4 Discussion

### 4.1 Co-application of B9 and vermicompost synergistically enhances litter decomposition in UGS soils

Urban green space (UGS) soils often accumulate large quantities of plant litter, whose decomposition is hindered by high cellulose and lignin content (Zhang et al., 2019; Liu et al., 2023). Within our experimental treatments, litter decomposition was greatest in the B9 + vermicompost group, while either component alone produced only modest improvements (Figure 1). Although cellulase activity in the vermicompost-only treatment appeared slightly higher than in the B9-only treatment, this difference was not statistically significant and likely reflects the baseline microbial stimulation provided by vermicompost rather than a true performance difference between the two treatments. This finding supports the idea that neither microbial inoculation nor organic amendments are independently sufficient for effective degradation of recalcitrant plant material in soils (Shaghaleh et al., 2023). The improved decomposition in the VB9 treatment can be attributed to functional complementarity: vermicompost provides labile carbon and nutrients that stimulate microbial growth, while B9 contributes cellulolytic enzymes that directly degrade structural polysaccharides.

This combination likely generates a positive feedback loop, in which the bioavailable carbon fractions liberated through enzymatic cellulose hydrolysis serve to augment microbial metabolic activity, consequently fueling the breakdown of other recalcitrant carbon forms (Talbot and Treseder, 2012). Similar synergistic effects have been reported in agricultural settings, where co-application of microbial inoculants and organic fertilizers promotes plant growth and nutrient cycling more effectively than either treatment alone (Chaturvedi and Pandey, 2021; Ullah et al., 2021; Sarathambal et al., 2024). However, such strategies have rarely been evaluated in UGS soils, which differ substantially in microbial diversity, disturbance history, and litter input patterns (Yan et al., 2018; dos Santos et al., 2020; Christel et al., 2023). Our findings suggest that co-applicating cellulolytic microbes and vermicompost may represent a practical strategy to accelerate organic matter turnover in UGSs, especially in soils with poor baseline microbial activity. This also highlights the potential of designing microbial–organic interaction-based restoration interventions for urban environments.

### 4.2 B9 harbors key genetic and enzymatic traits for efficient cellulose degradation

The enhanced decomposition observed in the VB9 treatment was consistent with B9’s functional traits. Genomic analysis revealed that B9 contains genes encoding key cellulolytic enzymes, including endoglucanases and *β*-glucosidases (Supplementary Figure 4). These enzymes act sequentially to depolymerize cellulose: endoglucanases cleave internal bonds of cellulose chains to produce cellobiose, while β-glucosidases convert cellobiose into glucose, removing end-product inhibition and facilitating sustained enzyme activity (Lin et al., 2016; Zhang et al., 2022). The presence and expression of these genes were confirmed by enzymatic assays in culture supernatants, which showed strong activity of both enzymes in fermentation broth (Figure 2a). These findings suggest a positive feedback mechanism during cellulose degradation, with glucose release energizing microbes while alleviating cellobiose-induced inhibition.

In addition to its cellulolytic capacity, B9 exhibited functional traits related to nitrogen transformation. Litter decomposition rate and cellulose degradation rate were positively correlated with AN and NH₄^+^-N levels (Figure 3). Further genomic prediction and experimental validation showed that B9 harbors DNRA potential, converting NO₃^−^-N to NH₄^+^-N (Supplementary Table 5; Supplementary Figure 5). Since NH₄^+^ is more easily absorbed and retained in soils, this metabolic trait likely enhanced nitrogen cycling and indirectly supported microbial activity in litter decomposition (Chen et al., 2014; Pandey et al., 2020). This dual role in carbon and nitrogen transformation reinforces B9’s ecological relevance.

In summary, B9 integrates carbon- and nitrogen-related functions to promote litter decomposition through complementary direct and indirect mechanisms. Rapid cellulose hydrolysis supplies soluble sugars that accelerate structural polysaccharide breakdown and stimulate heterotrophic microbial activity, thereby creating a more metabolically active decomposition environment (Talbot and Treseder, 2012; Wilhelm et al., 2021). These primary enzymatic effects interact with broader microbial processes, as B9 inoculation enhances bacterial richness and enriches cellulolytic and carbon-cycling taxa (Figure 4). Collectively, these community-level responses further reinforce decomposition, highlighting B9 as both an active degrader and a facilitator of microbial interactions that drive organic matter turnover.

### 4.3 B9 modifies microbial community composition and promotes functional shifts

Amplicon sequencing revealed that B9 inoculation combined with vermicompost significantly modified the structure of the soil bacterial community and increased the ACE index compared to the control (Figures 4b,c). This increase in bacterial diversity is advantageous for litter decomposition (Chiba et al., 2021). Taxonomic analysis showed an increased relative abundance of cellulolytic bacteria, such as *Bacillus* and *Sphingomonas* in B9 and VB9 treatments (Figure 4d). Such taxa are known for their roles in cellulose breakdown and organic matter decomposition (Tao et al., 2022; Dobrzyński et al., 2023). These shifts suggest that B9 may act as a keystone species that facilitates the colonization and activity of other cellulolytic bacterium and nutrient-cycling bacteria, potentially by modifying the local environment or through microbial interactions and accelerates litter decomposition.

Beyond taxonomic shifts, B9-mediated cellulose hydrolysis may further influence surrounding microbes by releasing soluble sugars that serve as readily utilizable carbon sources (Gunina and Kuzyakov, 2015). These labile substrates can stimulate the growth of heterotrophic bacteria and enhance overall microbial metabolic activity, providing an ecological mechanism through which cellulose degradation promotes broader microbial interactions in soil (Wilhelm et al., 2021; Zhou et al., 2025).

Functional prediction using FAPROTAX further supported these compositional changes, indicating elevated functional potential for cellulose degradation and nitrate reduction (Supplementary Figure 8). Particularly, the enhancement of DNRA-related pathways aligns with our observed increase in soil NH_4_^+^-N levels. Since NH_4_^+^ binds more readily to negatively charged soil particles, this shift also reduces nitrogen loss through leaching (Pandey et al., 2020; Wan et al., 2023). Moreover, correlation analysis revealed that the relative abundance of cellulolytic taxa was positively associated with NH_4_^+^-N content (Supplementary Figure 7), suggesting that nitrogen dynamics mediated by B9 played a role in shaping microbial communities and, consequently, decomposition efficiency.

### 4.4 Co-application enhances soil metabolic activity and nutrient availability

Non-targeted metabolomics revealed that the VB9 treatment significantly upregulated carbon metabolism pathways, including starch and sucrose metabolism (Figure 5). These labile carbon sources are critical for microbial energy supply and are functionally linked to cellulose degradation (Wilhelm et al., 2021). Their accumulation and subsequent degradation likely reflect an overall increase in microbial metabolic intensity and carbon cycling efficiency (Kelliher et al., 2005; Shi et al., 2018). These findings are supported by the observed enhancement of soil physicochemical properties: VB9 significantly increased soil available nitrogen (AN and NH_4_^+^-N), phosphorus (AP), and potassium (AK) levels (Table 1), which are closely tied to organic matter mineralization and microbial activity.

The relationships between microbial taxa, enzyme activity, and soil nutrient levels were further confirmed by Spearman correlation and Redundancy Analysis (RDA; Supplementary Figures 6, 7), showing that changes in microbial communities were strongly associated with NH_4_^+^-N, AN and AP dynamics. These results indicate that the microbial–organic synergy drives biochemical transformations and leads to measurable improvements in soil fertility, offering a practical pathway to enhance nutrient cycling in UGSs.

### 4.5 Implications and future directions

This study demonstrates that co-application of cellulolytic bacteria and vermicompost significantly enhances litter decomposition and nutrient cycling in UGS soils. By linking microbial inoculation, community shifts, metabolic pathways, and soil property improvements, we provide a mechanistic understanding of how microbial–organic interactions can be leveraged for ecological restoration. Importantly, our findings highlight the underexplored potential of combining cellulolytic functional strains with organic amendments in complex and heterogeneous urban soils.

Future research should assess the field-scale applicability of this approach across diverse UGS types and climates, investigate its long-term stability and environmental impacts, and explore the interaction of different microbial traits with various organic substrates. These steps are crucial to transforming microbe-amendment synergy from an experimental observation into a scalable solution for urban soil management.

## 5 Conclusion

This study demonstrates that *B. cereus* B9 enhances litter decomposition in UGS soils by promoting cellulose degradation and modifying microbial community structure. Genomic analysis identified genes encoding key cellulolytic enzymes-endoglucanase and *β*-glucosidase-which were functionally validated through fermentation broth enzyme assays. Combined with vermicompost, B9 significantly increased soil cellulase activity, enriched cellulolytic bacterium, and improved microbial diversity. This microbial shift was accompanied by higher NH_4_^+^-N content, likely mediated by DNRA-related pathways, and improved soil organic matter and nutrient availability. Metabolomic profiling further showed that B9 inoculation upregulated starch, sucrose, and nitrogen metabolism pathways, reinforcing its role in driving organic matter turnover. These findings highlight the potential of microbial-organic co-amendment as a sustainable strategy for improving soil fertility in UGSs. Future research should assess this approach’s long-term stability and applicability across diverse urban soil conditions.

## Data Availability

The complete genome sequence of Bacillus cereus B9 has been deposited in GenBank under accession number CP157848.1.
