# Supplementary material for: Enhancing soil fertility in urban green spaces via cellulolytic microbial-organic synergies
Source: Front Microbiol. 2026 Feb 5;17:1711396. doi: 10.3389/fmicb.2026.1711396 (PMC12916661; doi:10.3389/fmicb.2026.1711396)
Supplement: Supplementary file 1 [file Table_1.docx]

Supplementary information for

**Enhancing Soil Fertility in Urban Green Spaces via Cellulolytic Microbial–Organic Synergies**

Zhaofeng Xu^1^, Jiawei Dai^1^, Ning Yang^1^, Yongjie Fan^1^, Xin Shan^1^, Yuting Diao^1^, Xiaocui Pan^1,2^, Lei Zhao^3^, Jiahui Zhao^1^, Meiqi Ma^1^, Xiang Li^1^, Ming Xiao^1^, Junmin Pei^1*^

^1^ *College of Life Sciences, Shanghai Normal University, Shanghai 200234, China*

^2^ *School of Life Sciences, Taizhou University, Taizhou, Zhejiang Province 318000, China*

^3^ *Shanghai Institute of Quality Inspection and Technical Research, Shanghai 200233, China*

^*^Corresponding authors:

Dr. Junmin Pei; Tel: +86-(0)-21-64321022; Fax: +86-(0)-21-65642468; E-mail: [junminpei@shnu.edu.cn](mailto:junminpei@shnu.edu.cn)

**Contents of this file**

Supplementary Figures 1 to 9

Supplementary Tables 1 to 5


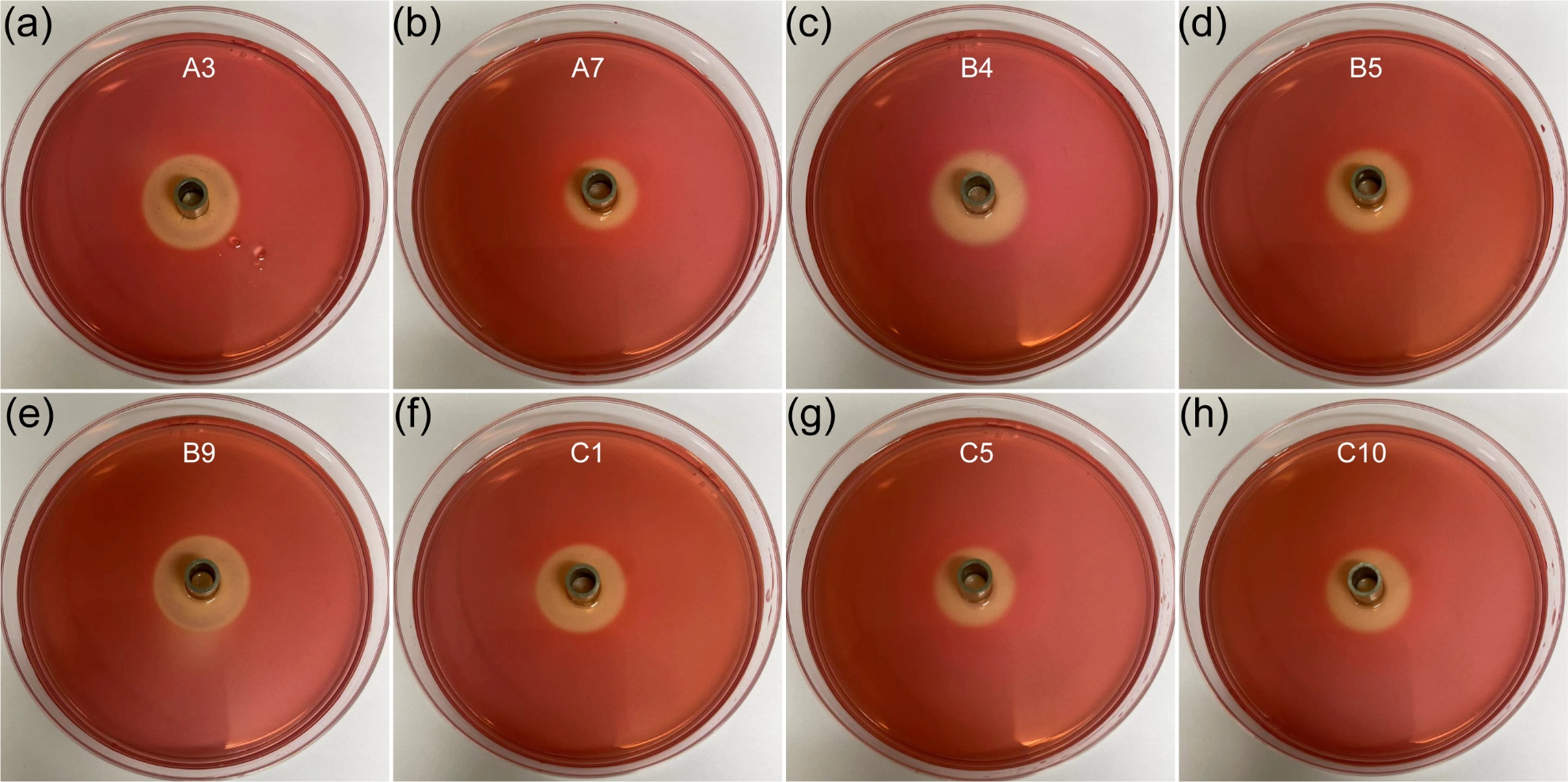


**Supplementary Figure 1. Congo red–CMC plate assay for screening cellulolytic bacteria.** Clear halos surrounding colonies of isolates A3 (a), A7 (b), B4 (c), B5 (d), B9 (e), C1 (f), C5 (g), and C10 (h) manifested as zones with a loss of Congo red staining in the CMC medium, indicating cellulose degradation.


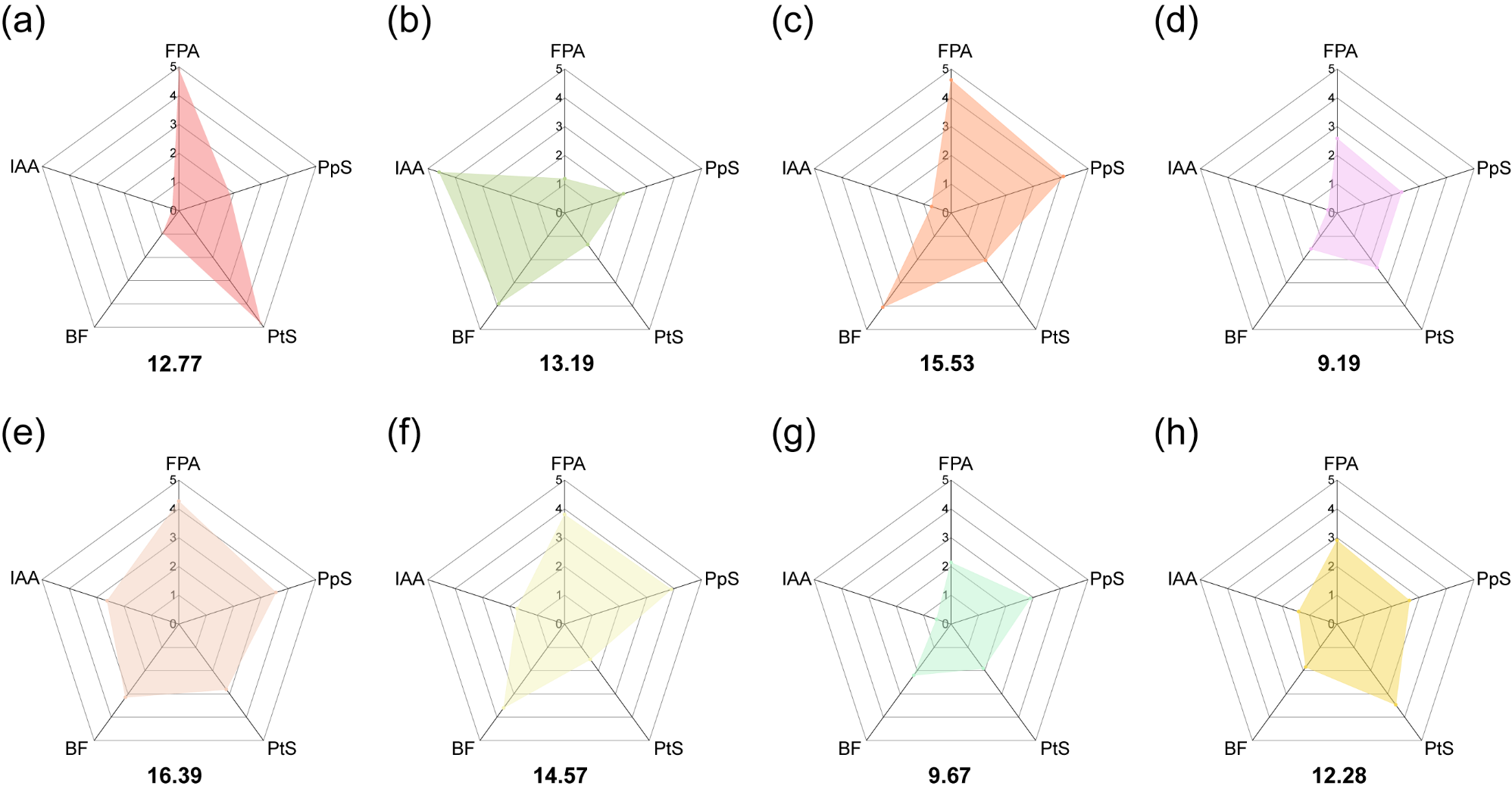


**Supplementary Figure 2. Radar chart illustrating functional trait scores of the eight isolated strains.** This chart compares five biochemical and plant growth–promoting traits across the tested bacterial strains A3 (a), A7 (b), B4 (c), B5 (d), B9 (e), C1 (f), C5 (g), and C10 (h): filter paper activity (FPA), phosphate solubilization (PpS), potassium solubilization (PtS), biofilm formation (BF), and indole-3-acetic acid (IAA) production. The value beneath each panel represents the corresponding strain's overall score. Details of the scoring criteria are provided in **Supplementary Table 2**.


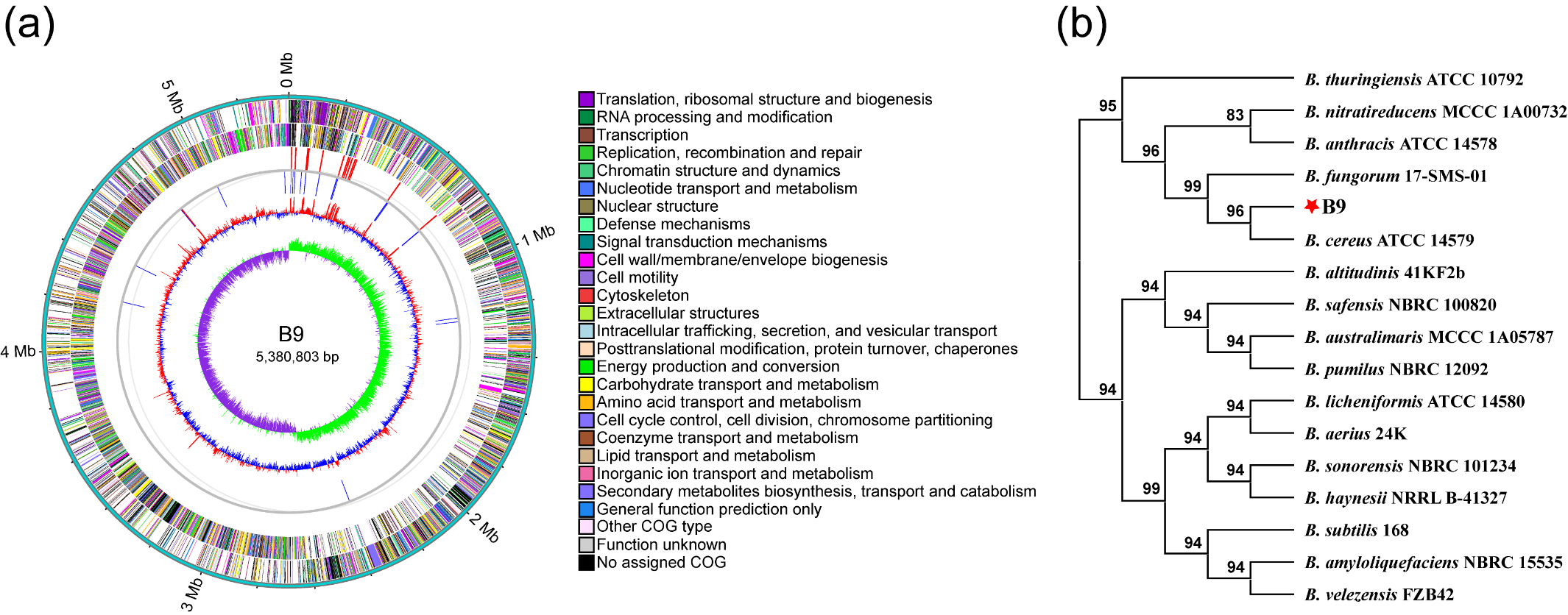


**Supplementary Figure 3.** **Genomic characteristics and phylogenetic tree of B9 strain.** The circular map of B9’s chromosome (a), the outermost circle of the circular diagram represents the genome size, with each tick representing 0.2 Mb. The second and third circles represent coding sequences (CDS) on the forward and reverse strands, respectively. Different colors indicate different functional classifications of CDS based on Clusters of Orthologous Groups (COG). The fourth and fifth circles represent rRNA and tRNA on the forward and reverse strands, with rRNA shown in red and tRNA in blue. The sixth circle represents the GC content, where outward red portions indicate regions with higher GC content compared to the average GC content of the whole genome. The peak height represents the difference from the average GC content, with higher peaks indicating larger differences. Inward blue portions indicate regions with lower GC content compared to the average GC content of the whole genome, with higher peaks representing larger differences. The innermost circle represents the GC skew value, calculated as (G - C) / (G + C). Biologically, positive values indicate a bias towards transcription of CDS on the forward strand, represented by green, while negative values indicate a bias towards transcription of CDS on the reverse strand, represented by purple. The phylogenetic tree (b) demonstrates B9's position in relation to other strains of *Bacillus spp.* This tree was constructed based on 16S rRNA gene alignments, with sequences aligned using ClustalW and a maximum-likelihood tree generated by MEGA-Ⅹ. A total of 1000 replications were conducted for bootstrap testing, with bootstrap values displayed at each node.


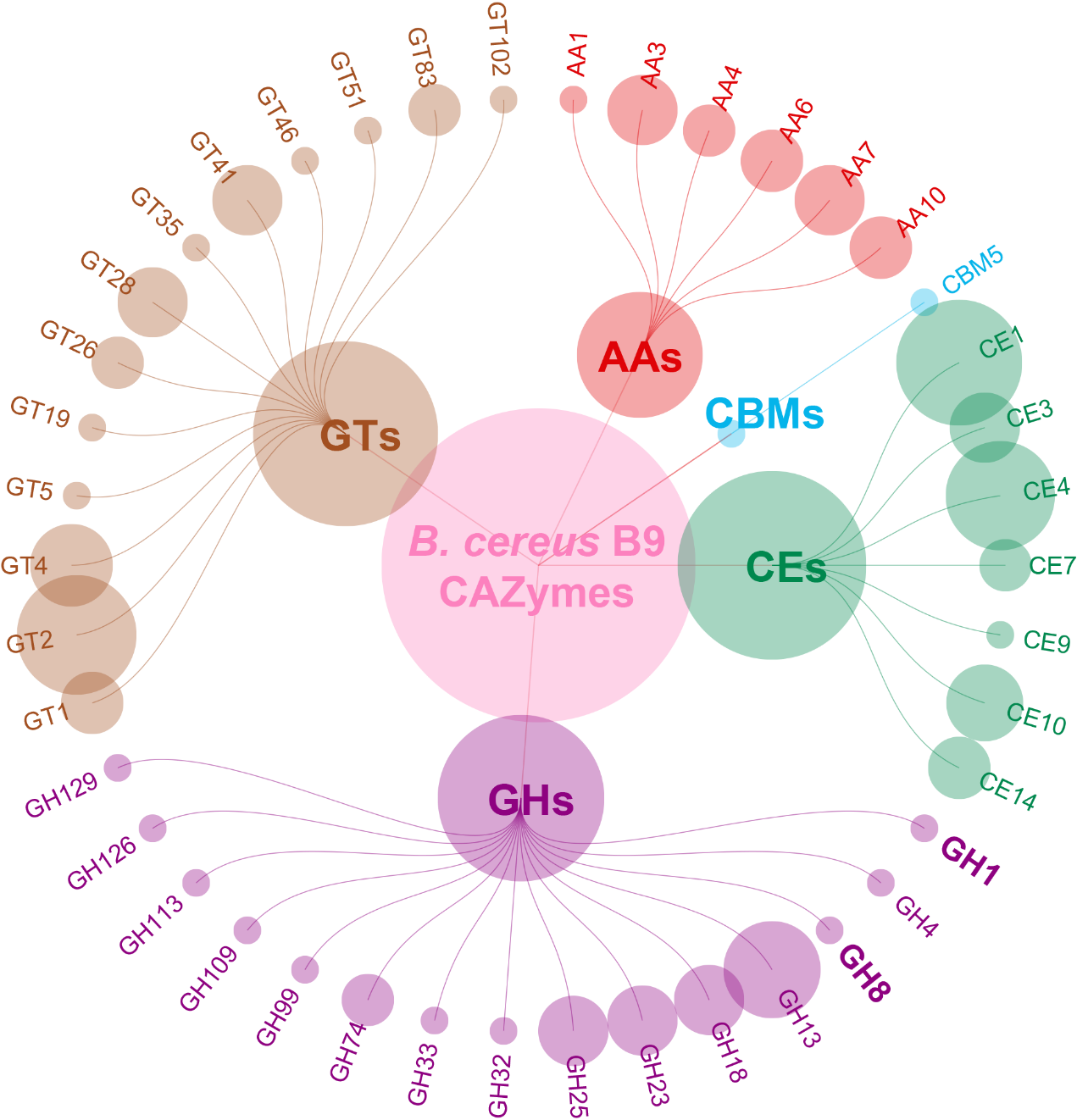


**Supplementary Figure 4. Classification annotation of carbohydrate-active enzymes for *B. cereus* B9.** The outer ring shows the different enzyme families within each taxonomic category, and the size of the circle represents the number of genes annotated by each family. GHs: Glycoside Hydrolases; GTs: Glycosyl Transferases; CEs: Carbohydrate Esterases; AAs: Auxiliary Activities; CBMs: Carbohydrate-Binding Modules.


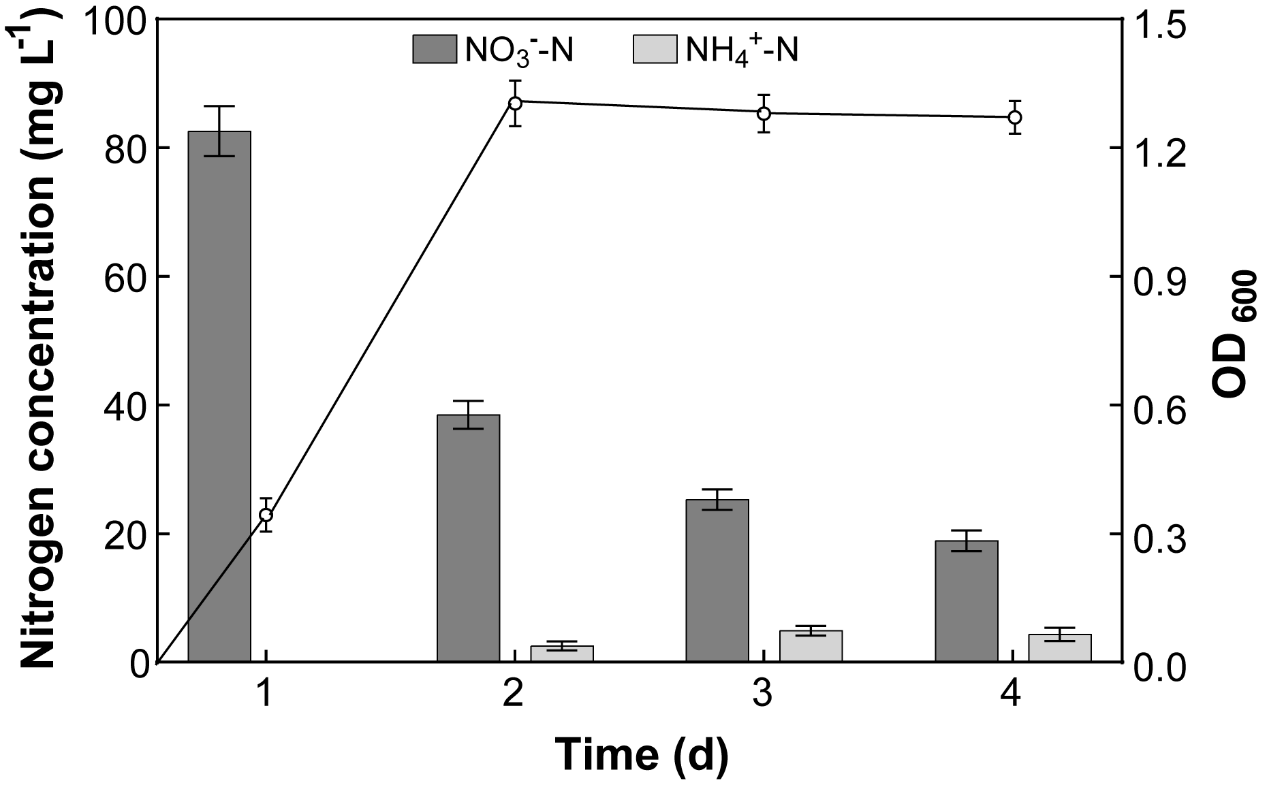


**Supplementary Figure 5. Characteristic for nitrate transformation of *B. cereus* B9.** The concentrations of NO_3_^-^-N and NH_4_^+^-N in denitrification medium are depicted on the left y-axis, while the cell concentration (○) of *B. cereus* B9 in the medium is shown on the right y-axis. Data represent means of three replicates, with error bars indicating standard deviations.


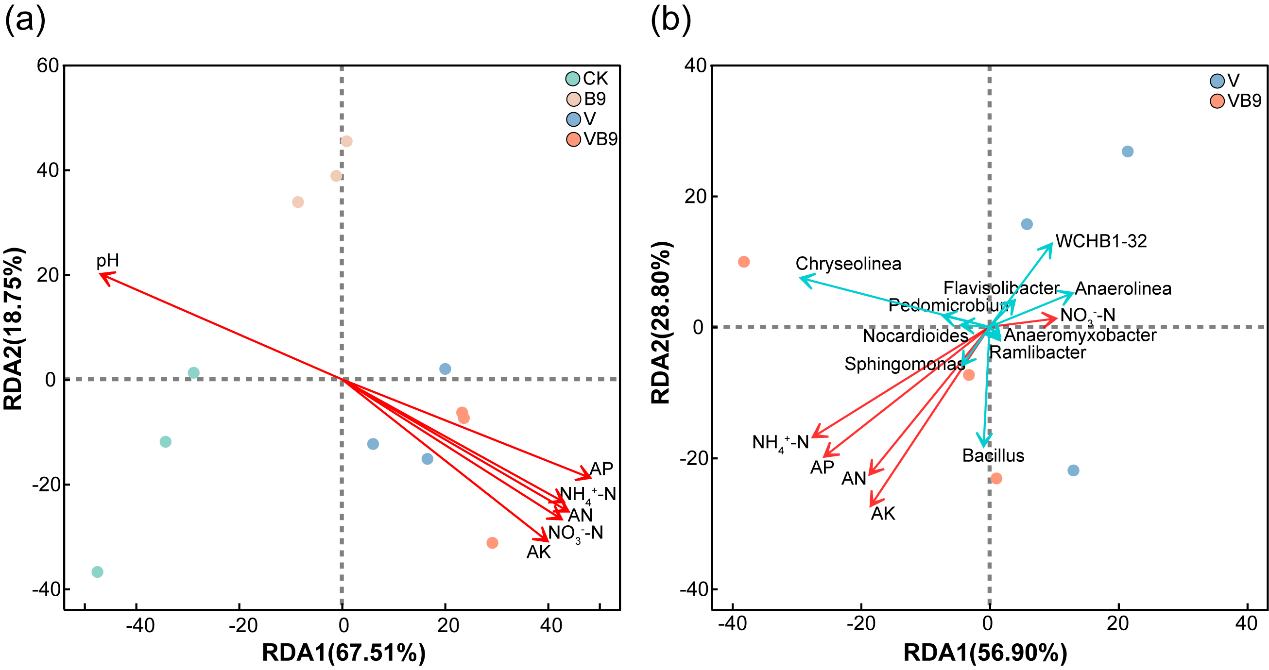


**Supplementary Figure 6. Redundancy analysis of soil chemistry properties and bacterial community composition.** (a) was derived from four soil groups, while (b) was derived from soils amended with vermicompost (with or without B9). Details of the treatments are provided in **Figure 1**. Dots of different colors represent sample groups under different treatments. Red arrows denote quantitative environmental factors, while light blue arrows represent species. The length of the arrows indicates the extent of influence of environmental factors on the distribution of species data across different sample groups (i.e., explanatory power). The angles between environmental factor arrows reflect their correlations: acute angles indicate positive correlations, obtuse angles negative correlations, and right angles no correlation. The distance from the projection point to the origin signifies the relative influence of environmental factors on the sample community distribution. Additionally, whether the direction of the point and the arrow align indicates positive or negative correlation.


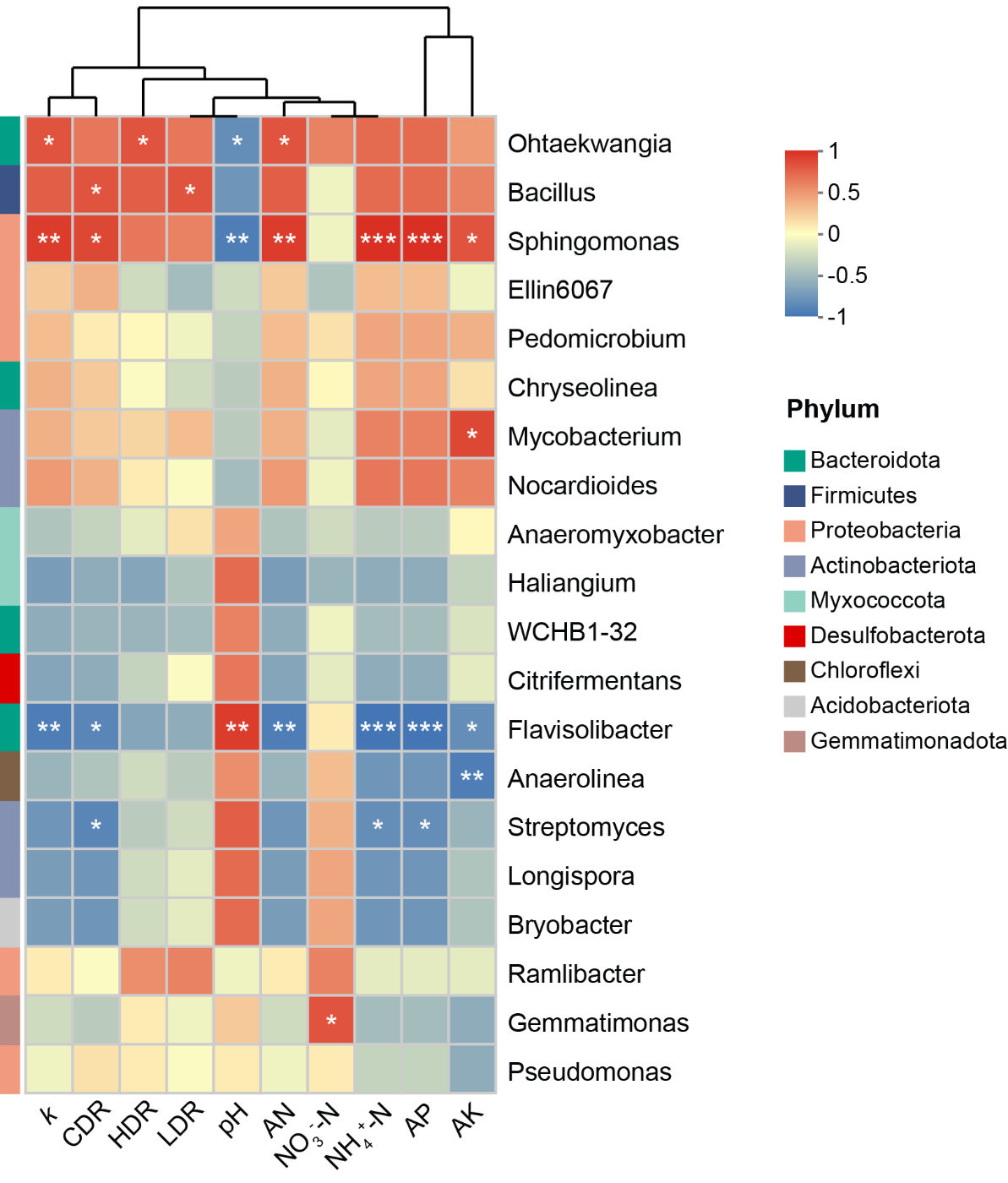


**Supplementary Figure 7. The Correlation Heatmap diagram shows the correlation between species and environmental factors.** Spearman correlation analysis among bacterial genera, soil chemical properties and litter decomposition parameters. *k*, litter decomposition rate constant; CDR, cellulose degradation rate; LDR, lignin degradation rate; HDR, hemicellulose degradation rate. The legend on the right is the color range of different R values. The different colors represent the classification of species at the phylum level. * *p* < 0.05, ** < *p* < 0.01, *** *p* < 0.001.


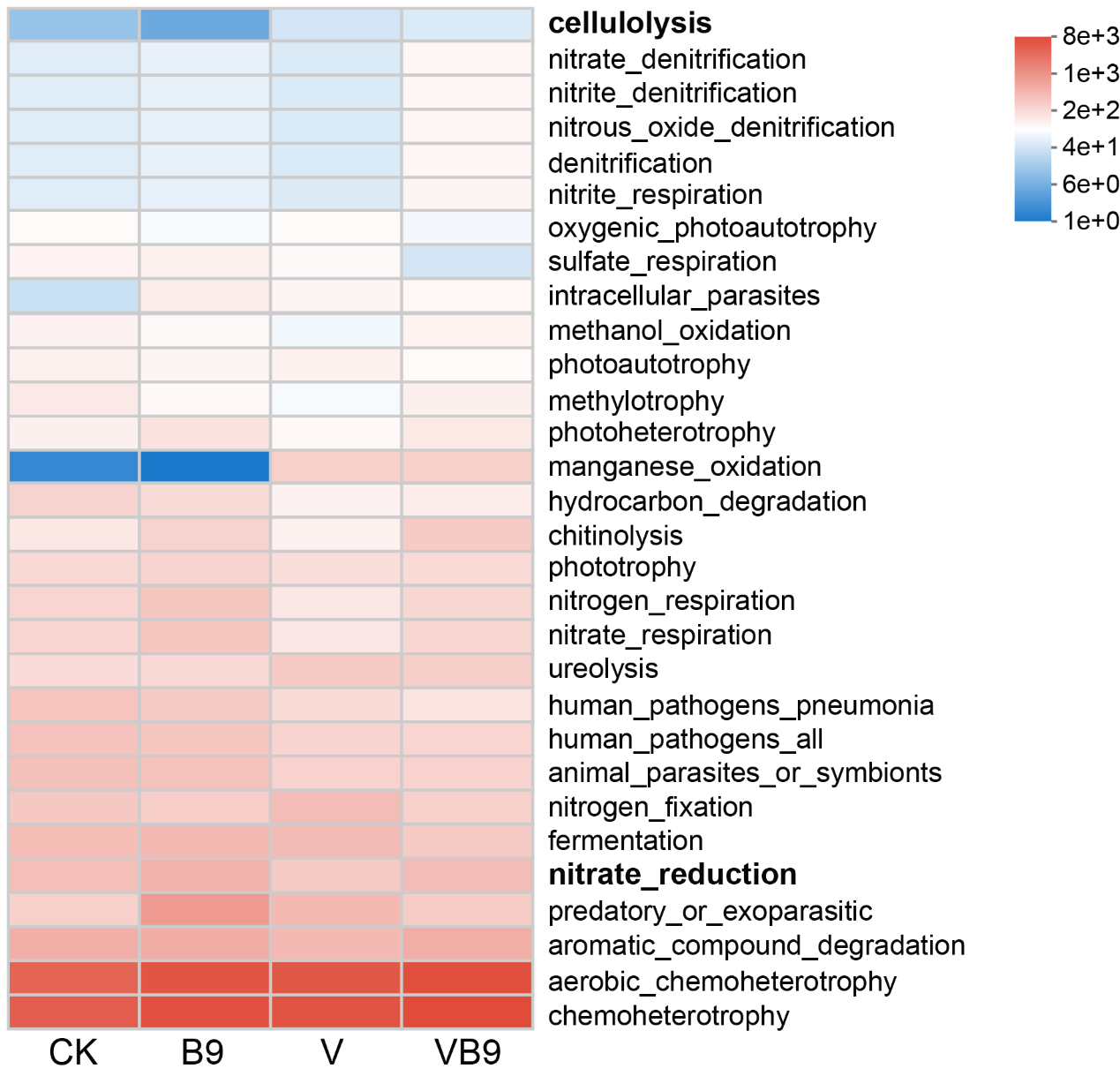


**Supplementary Figure 8. Differences in bacterial community function in soil under different treatments.** FAPROTAX was used to predict the mean proportions of functional profiles of bacterial communities in each group. Details of the treatments are provided in **Figure 1**.


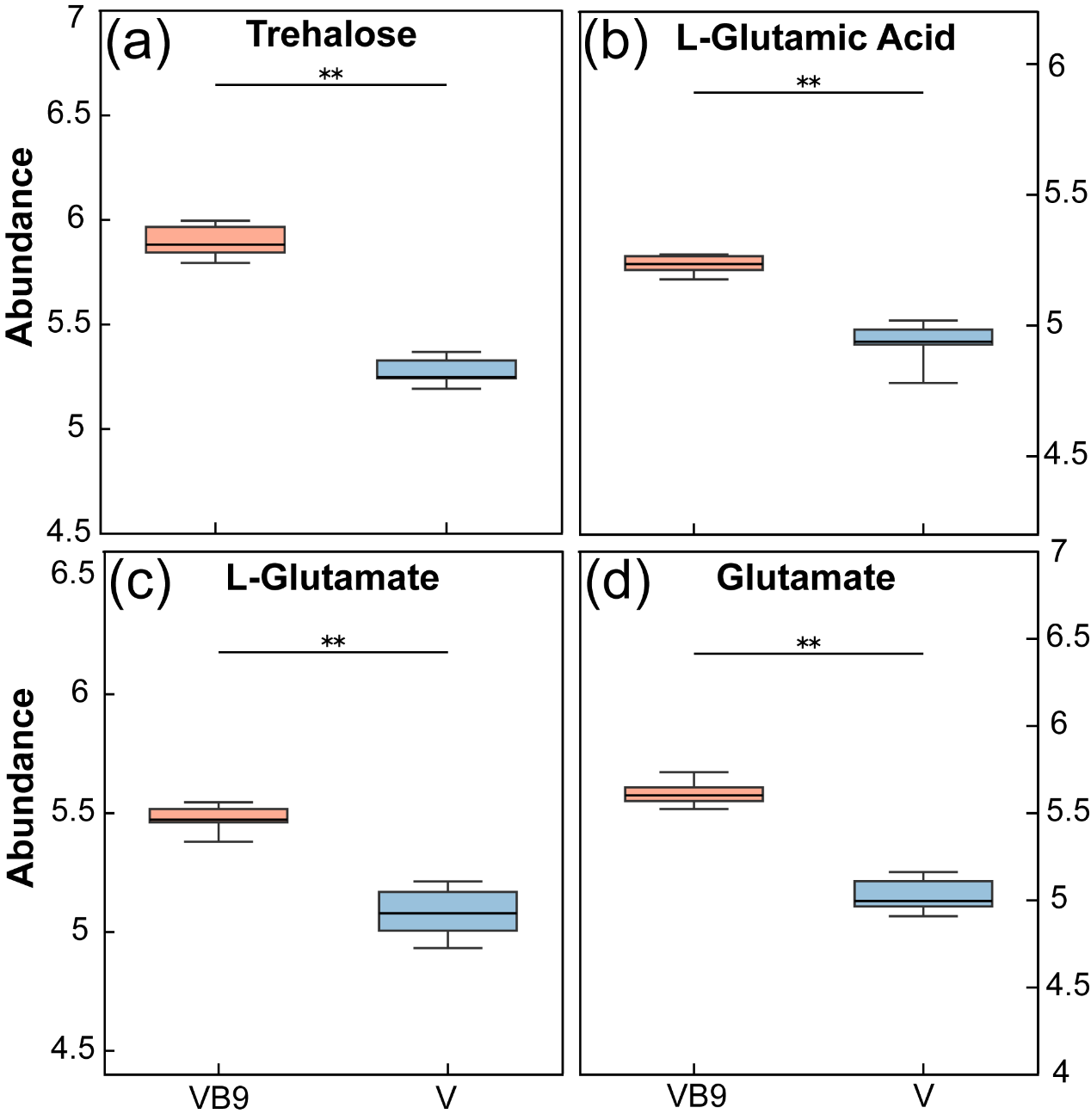


**Supplementary Figure 9. Differential metabolites in soils in the V and VB9 groups.** Metabolites were detected in the V and VB9 groups with significant differences in nitrogen metabolism and sucrose and starch metabolism pathways. Details of the treatments are provided in **Figure 1**. ** VIP_pred_OPLS-DA > 1, *p* < 0.01.

**Supplementary Table 1.** Physicochemical properties of the soil used in the experiments.

| Properties | Silt（%） | Clay（%） | Sand（%） | pH | AN  (mg kg^-1^) | AP  (mg kg^-1^) | AK  (mg kg^-1^) |
| --- | --- | --- | --- | --- | --- | --- | --- |
| Soil | 67 | 14 | 19 | 8.25±0.03 | 36.88±0.70 | 13.22±0.44 | 124.6±6.02 |

Note: pH, potential of hydrogen; AN, alkali-hydrolysable nitrogen; AP, available phosphorus; AK, available potassium. Data are represented as means ± SD (n = 5).

**Supplementary Table 2.** Scoring criteria for the biochemical and plant growth–promoting traits of the isolated strains.

| FPA (U mL^-1^) | PpS (mg L^-1^) | PtS (mg L^-1^) | BF (OD_595_) | IAA (mg L^-1^) | Score |
| --- | --- | --- | --- | --- | --- |
| 0-2 | 0-0.2 | 0-10 | 0-0.2 | 0-6 | 1 |
| 2-4 | 0.2-0.4 | 10-20 | 0.2-0.4 | 6-12 | 2 |
| 4-6 | 0.4-0.6 | 20-30 | 0.4-0.6 | 12-18 | 3 |
| 6-8 | 0.6-0.8 | 30-40 | 0.6-0.8 | 18-24 | 4 |
| 8-10 | 0.8-1 | 40-50 | 0.8-1 | 24-30 | 5 |

Note: FPA, filter paper activity; PpS, phosphate solubilization; PtS, potassium solubilization; BF, biofilm formation; IAA, indole-3-acetic acid production.

**Supplementary Table 3.** Linear regression analysis of litter decomposition rate constants *k* and lignocellulose degradation rates.

|  | **Unstandardized Coefficients** | | **Standardized Coefficients** | ***t*** | ***p*** | **Collinearity Diagnostics** | |
| --- | --- | --- | --- | --- | --- | --- | --- |
|  | ***B*** | **SE** | ***Beta*** |  |  | **VIF** | **Tolerance** |
| **Constant** | -0.001 | 0.003 | - | -0.235 | 0.822 | - | - |
| **CDR** | 0.013 | 0.003 | 0.896 | 4.223 | 0.006** | 1.130 | 0.885 |
| **HDR** | 0.002 | 0.005 | 0.078 | 0.324 | 0.757 | 1.458 | 0.686 |
| **LDR** | 0.006 | 0.006 | 0.276 | 1.097 | 0.315 | 1.595 | 0.627 |
| **Sample size** | 10 | | | | | | |
| ***R* ^2^** | 0.761 | | | | | | |
| **adjust *R* ^2^** | 0.641 | | | | | | |
| ***F*** | *F* (3,6) =6.367, *p*=0.027 | | | | | | |
| **D-W** | 2.198 | | | | | | |
| Note: Analysis of data from groups V and VB9. Dependent Variable = *k* (Decomposition rate constants of litter); Independent Variable = CDR, HDR and LDR (Degradation rates of cellulose, hemicellulose and lignin). *B* is the regression coefficient for each independent variable. *p*<0.05 indicates a significant correlation between the independent variable and the dependent variable. *R* ^2^ represents the goodness of fit for the model, with values approaching 1 indicating a better fit. In the model, VIF < 5 and Tolerance > 0.2 mean that there is no collinearity problem. The D-W value is close to 2, thus indicating that the model has no autocorrelation and there is no correlation among the sample data. * *p*<0.05 ** *p*<0.01. | | | | | | | |

**Supplementary Table 4.** The BLAST results of the amino acid sequence encoded by gene2645 in UniProtKB.

| Organism | Gene Name | Entry Name | Protein Name | Length (AA) | Homology (%) |
| --- | --- | --- | --- | --- | --- |
| *Bacillus cereus* (strain ATCC 14579) | BC_2682 | Q81CR5_BACCR | Glucanase, 3.2.1.- | 453 | 98.7 |
| *Bacillus anthracis* | GBAA_2673 | A0A6L8PFN9_BACAN | Glucanase, 3.2.1.- | 453 | 97.4 |
| *Bacillus* sp. (strain KSM-330) | - | GUN_BACSZ | Endoglucanase, 3.2.1.4 | 463 | 77.3 |
| *Paenibacillus radicis* | GCM10010918_03530 | A0A917GQ25_9BACL | Glucanase, 3.2.1.- | 654 | 73.7 |
| *Bacillus* sp. FJAT-26390 | A7975_17750 | A0A1B8W487_9BACI | Glucanase, 3.2.1.- | 646 | 74 |
| *Paenibacillus lutimineralis* | EI981_15705 | A0A3Q9I9H1_9BACL | Glucanase, 3.2.1.- | 586 | 72.4 |
| *Paenibacillus* sp. GSMTC-2017 | I6N90_24300 | A0A931ND77_9BACL | Glucanase, 3.2.1.- | 796 | 72.7 |
| *Paenibacillus* sp. oral taxon 786 str. D14 | POTG_00496 | C6IWG9_9BACL | Glucanase, 3.2.1.- | 556 | 71.8 |
| *Paenibacillus anaericanus* | EJP82_13755 | A0A3S1C8W3_9BACL | Glucanase, 3.2.1.- | 657 | 73.2 |
| *Paenibacillus antarcticus* | PBAT_16860 | A0A162MFX8_9BACL | Glucanase, 3.2.1.- | 659 | 73.2 |

**Supplementary Table 5.** Genes related to nitrogen metabolism in the B9 genome.

| **Gene ID** | **Gene Name** | **Gene function** |
| --- | --- | --- |
| gene2085; gene2086; gene2087; gene2088 | ***narG****;* ***narH****;* ***narJ****;* ***narI*** | nitrate reductase |
| gene2095 | *narK* | nitrate transporter NarK |
| gene1425 | *nirA* | ferredoxin--nitrite reductase |
| gene2102 | ***nirB*** | NADPH-nitrite reductase large subunit |
| gene1311 | *nirC* | formate/nitrite transporter family protein |
| gene2101 | ***nirD*** | nitrite reductase |
| gene1369 | *npd* | nitronate monooxygenase |
| gene3488 | *hcp* | hydroxylamine reductase |
| gene0529 | *gltB* | glutamate synthase |
| gene1490 | *gudB* | NAD-specific glutamate dehydrogenase |
| gene3743 | *glnA* | glutamine synthetase |
